# Inactivated COVID-19 vaccine induced acute stroke-like focal neurologic symptoms: a case series

**DOI:** 10.1186/s12883-022-02739-6

**Published:** 2022-06-07

**Authors:** Duangnapa Roongpiboonsopit, Chichaya Nithisathienchai, Wasan Akarathanawat, Krittanon Lertutsahakul, Jarturon Tantivattana, Anand Viswanathan, Nijasri Charnnarong Suwanwela

**Affiliations:** 1 Division of Neurology, Department of Medicine, Faculty of Medicine, Naresuan University, 99 Moo 9, Tha Pho, Mueang, Phitsanulok, 65000 Thailand; 2 Division of Neurology, Department of Medicine, Faculty of Medicine, Chulalongkorn University, Bangkok, Thailand; 3 Chulalongkorn Comprehensive Stroke Center, King Chulalongkorn Memorial Hospital, Bangkok, Thailand; 4 Department of Radiology, Faculty of Medicine, Chulalongkorn University, Bangkok, Thailand; 5 Department of Neurology, Harvard Medical School, Massachusetts General Hospital, Boston, MA USA; 6 Chula Neuroscience Center, King Chulalongkorn Memorial Hospital, Bangkok, Thailand

**Keywords:** Neurological complication, Inactivated COVID-19 vaccine, Stroke-like symptoms, Case series

## Abstract

**Background:**

A subgroup of individuals experienced stroke-like symptoms after receiving an inactivated COVID-19 vaccine. We present clinical characteristics, neuroimaging, and outcome of these patients.

**Methods:**

Medical personals who had neurological symptoms after receiving inactivated COVID-19 vaccine were enrolled. Clinical, laboratory investigation and neuroimaging were collected. Subjects were prospectively followed-up on clinical and neuroimaging to detect brain parenchymal or cerebrovascular abnormality.

**Results:**

Nineteen out of 385 subjects (4.9%) developed neurological symptoms after vaccination. There was a female predominance (89.5%) with mean age of 34 ± 7.5 years. Majority of patients (52.6%) had symptoms within 60 min after vaccination. The most common neurological symptoms were numbness (94.7%) followed by headache (52.6%) and weakness (47.4%). The most common neurological signs were sensory deficit (79%) followed by motor weakness (52.6%) and tongue deviation (26.3%). Recurrent headache was observed in most patients (89.5%) during followed up. Serial brain imaging was done in all patients with median follow-up interval of 18 days. There was no evidence of acute brain infarction in any of the patients, 84.2% had no vascular abnormality, 15.8% had transient focal narrowing of cerebral vessels. Outcome was favorable, modified ranking scale 0–1 for all patients at 4 weeks after vaccination.

**Conclusions:**

Transient focal neurological symptoms and deficits can be found after COVID-19 vaccination. However, benefit to stop COVID-19 pandemic by vaccination is outweighed by these seemingly reversible side effects. The pathophysiology underlined these phenomena should be further investigated.

**Supplementary Information:**

The online version contains supplementary material available at 10.1186/s12883-022-02739-6.

## Introduction

In the corona virus disease 2019 (COVID-19) pandemic era, vaccination is an important strategy to stop disease spread as well as decrease complications and mortality from the corona virus infection. Coronavac manufactured by Sinovac Life Sciences Co., Ltd, Beijing, China which is an inactivated virus vaccine has been approved by Food and Drug Administration for emergency use in Thailand and recently approved by the World Health Organization [1]. Two-dose schedule given intramuscularly with an interval of 2–4 weeks was initial recommend. In phase I/II and III clinical trial of the vaccine, very small number of patients experienced headaches or had neurological complications such as acute disseminated encephalomyelitis, clinically isolated syndrome were reported as an adverse reactions [2–5]. At our hospital, the COVID-19 vaccination program was started on 16^th^ April 2021. Medical personals were given priority for vaccination. Among them, many developed focal neurological symptoms and were admitted to our hospital. In this series, we describe clinical characteristics, brain imaging and outcome of patients who developed acute neurological syndrome after inactivated virus COVID-19 vaccination.

## Methods

This is a retrospective, single-center study. In this case series, we present data that were collected in a period from 16 April 2021 to 23 April 2021 at Naresuan University Hospital, Phitsanulok, Thailand. A total of 385 medical professional received the first dose of CoronaVac vaccine (inactivated virus COVID-19 vaccine). Individuals who developed acute neurological symptoms after vaccination were recruit. All objective deficits were examined, confirmed and reported by vascular neurologist (DR). Baseline characteristics, clinical, laboratory investigation and neuroimaging were collected. Brain computed tomography (CT), magnetic resonance imaging (MRI), and magnetic resonance angiography (MRA) without contrast injection (time-of-flight technique, TOF) or CT angiography was performed at the time of presentation. The patients were scanned at a Brilliance 64-slice CT scan (Philips Healthcare, Eindhoven, the Netherlands) and at a Ingenia 1.5 T MRI scan (Philips Medical system, Best, the Netherlands). MR sequences included a axial T1-weighted (slice thickness, 5 mm; repetition time, 450 ms; echo time, 15 ms; flip angle 69°), axial T2-weighted (slice thickness, 5 mm; repetition time, 4949.0 ms; echo time, 100 ms; flip angle 90°), axial fluid-attenuated inversion recovery (slice thickness, 5 mm; repetition time, 11,000 ms; echo time, 140 ms; flip angle 90°), diffusion-weighted imaging (b1000, slice thickness, 5 mm; repetition time, 4199.8 ms; echo time, 116.7 ms; flip angle 90°), TOF (slice thickness, 1.4 mm; repetition time, 25 ms; echo time, 6.9 ms, flip 20°), gradient recalled echo (slice thickness, 5 mm; repetition time, 643.4 ms; echo time, 13.8 ms; flip angle 18°). Clinical and brain imaging were followed-up until the patient had clinical fully recovery. All images were reviewed by 3 neurointerventionists (WA, KL, JT).

### Statistical analysis

Baseline characteristics, clinical, neuroimaging characteristics were described as appropriate. Categorical variables were described as frequency and percentage. Normally distributed quantitative variables were described as average and standard variation, and skewed distributed data were described as median and interquartile range. Kaplan–Meier plot with significant testing by log-rank test were used to determine type of focal neurological symptoms associated with symptom- free survival. Two tailed p-value of less than 0.05 were considered significant. Stata software (version 14.0, Stata corp., College Station, TX) were used for all analyses.

## Results

Among 385 subjects (female 68.3% and male 31.7%) who were vaccinated with Coronavac vaccine, 19 subjects developed acute neurological symptoms (4.9%). Baseline characteristics show in Table 1. Clinical, neuroimaging features, and outcome of the entire cohort show in Table 2.

**Table 1:** Baseline characteristics of patients with neurological symptoms after vaccinating inactivated COVID-19 vaccine

**Characteristics**	*n* = 19
Age, year, mean (SD)	34 (7.5)
Gender, Female	17 (89.5)
Underlying disease	7 (36.8)
Allergic rhinitis	2 (10.5)
Skin allergy	1 (5.2)
Asthma	1 (5.2)
Polycystic ovary syndrome	1 (5.2)
Chocolate cyst	1 (5.2)
Systemic lupus erythematosus	1(5.2)
Associated trigger	
Caffeine	8 (42.1)
Vasoconstrictive drugs	0
Contraceptive medications	3 (15.8)
Prior headache disorders	9 (47%)
Smoking	1 (5.2)
Recreational drug use	0
Drug allergic reaction	3 (15.8)
Psychiatric disorders including panic, hyperventilation and anxiety	0
History of food allergy	1 (5.2)
Body mass index, mean (SD)	21.3 (2.5)
Systolic Blood pressure, mmHg, mean (SD)	123.5 (9.8)
Diastolic Blood pressure, mmHg, mean (SD)	73.4 (10.6)
Heart rate, beat per minute, mean (SD)	89.8 (13.2)

*Abbreviation*: *SD* Standard deviation

**Table 2:** Clinical, neuroimaging features and outcome of patients with neurological symptoms after vaccinating inactivated COVID-19 vaccine

**Characteristics**	*n* = 19
Neurological symptoms, n (%)	
Unilateral numbness	18 (94.7)
Headache at the initial presentation	10 (52.6)
Headache during the course	17 (89.5)
Unilateral weakness	9 (47.4)
Nausea /vomiting	4 (21.1)
Perioral numbness	3 (15.8)
Neurological deficits, n (%)	
Sensory deficits	15 (79)
Motor deficits	10 (52.6)
Tongue deviation	5 (26.3)
Time of neurological symptom after vaccination, mins (median, IQR)	55 (21,661)
Time to complete recovery	
< 24 h	0 (0)
24–72 h	1 (5.3)
72 h -1 week	3 (15.8)
1–2 weeks	8 (42.1)
2–4 weeks	2 (10.5)
> 4 weeks	5 (26.3)
**Brain imaging**	
Initial CT brain with normal findings, n (%)	15 (78)
Time from symptom onset to CT scan, hr, median (IQR)	6.04 (2.3,13.5)
Initial MRI brain with normal findings, n (%), n (%)	19 (100)
Time from symptom onset to MRI scan, hr, median (IQR)	2.56 (1.6, 7.5)
Non-invasive vascular imaging (CTA/MRA), n (%)	19 (100)
No vascular abnormality	16 (84.2)
Dynamic angiographic change in luminal narrowing, n (%)	3 (15.8)
**Outcomes**	
NIHSS	
Initial, median (IQR)	1 (1,2.5)
at 2-week, median (IQR)	0 (0,0)
at 4-week, median (IQR)	0 (0,0.5)
mRS	
Initial, median (IQR)	1 (0.5,2)
at 2-week, median (IQR)	0 (0,1)
at 4-week, median (IQR)	0 (0,1)

*Abbreviation*: *CT* computed tomography, *IQR* inter quartile range, *MRI* magnetic resonance imaging, *mRS* modified rankin scale, *NIHSS* national institute of health stroke scale

### Clinical features

**Fig. 1:**
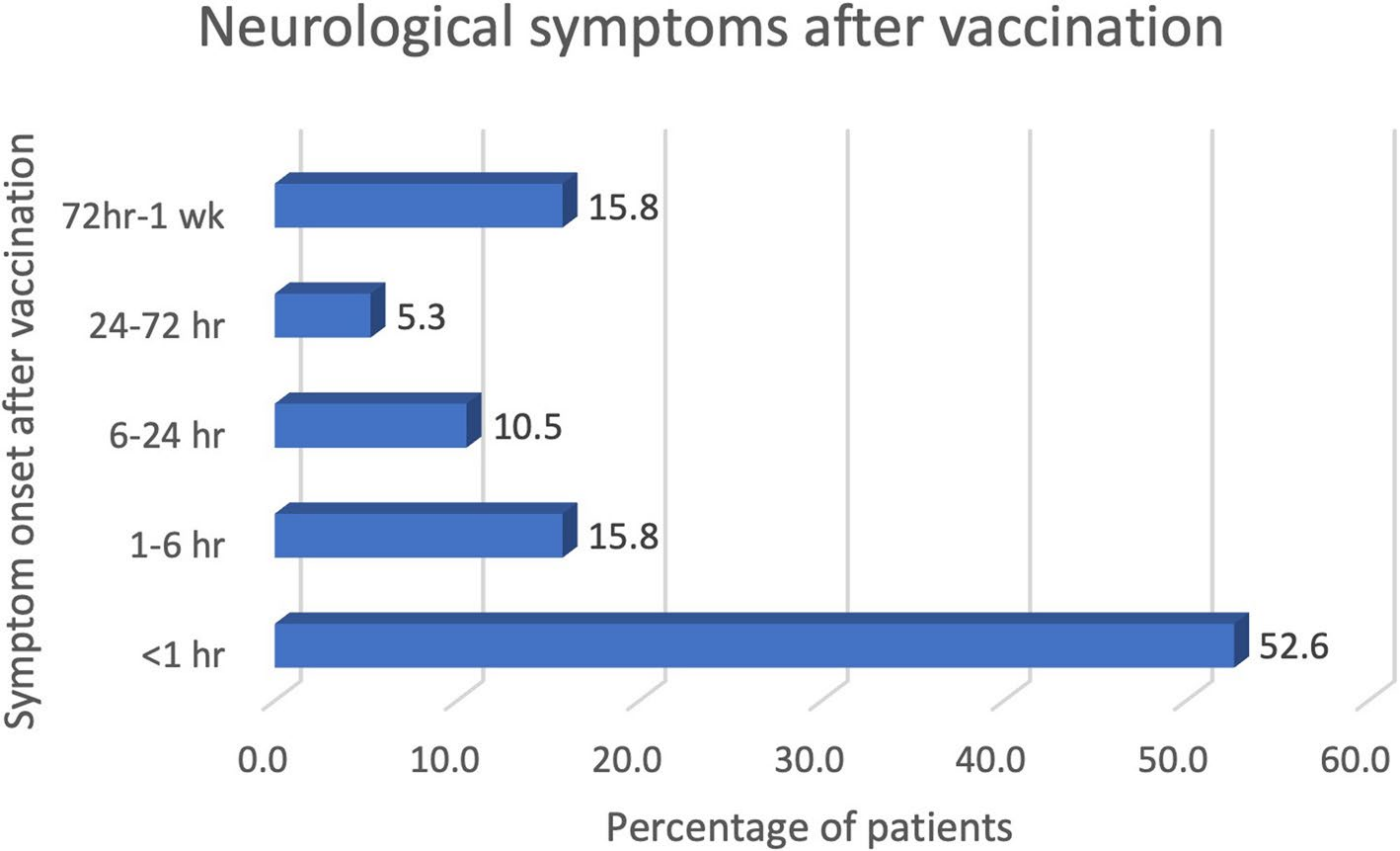
Onset of neurological symptom after vaccination

Among 19 patients, the mean age was 34 years (range, 23–46 years). Woman composed of 89% of the cohort. Majority of the patients (52.6%) had sudden neurological symptoms within 60 min after vaccination, median 55 min (IQR 21, 661 min) (Fig. 1). Seven patients (37%) developed the symptoms while under observation after vaccination and 12 (63%) experienced the abnormalities later and had to come back to the emergency room. All patients were initially evaluated and managed under our acute stroke protocol. The most common presentations were unilateral sensory symptom (94.7%) followed by headache (52.6%), weakness (47.4%), nausea vomiting (21.1%) and perioral numbness (15.8%). Most patients (79%) reported a progression of abnormal sensory symptoms within 30 min (IQR 15, 60 min). The most common neurological signs were sensory deficits (79%). Seven patients (36.8%) reported hemiparesthesia whereas 8 patients (42.1%) had paresthesia at one limb. Half of patients (50%) had sensory deficit in the opposite side of vaccination and other half had sensory deficit in the same side of vaccination. No local signs of inflammation were detected in patients who had symptoms ipsilateral to the side of vaccination. Motor weakness with pyramidal weakness pattern was found in 10 cases (52.6%), of these, 2 cases had monoparesis, 7 cases had hemiparesis and 1 case had triparesis, and in 26.3% tongue deviation was observed. The symptoms can be classified in 3 main groups 1) Isolated unilateral sensory symptoms (7 patients, 36.8%) 2) Sensory and motor symptom (11 patients, 57.9%) 3) Isolated severe headache symptom (1 patient, 5.3%). Most patients experienced recurrent headache (89.5%) over day 0 to day 8 after vaccination, of these 82.5% reported new-ever headache. For new-ever headache, the patients reported they have never experienced headache character as they reported before. Common headache feature was bandlike headache at small area of occiput or vertex with average pain score of 6/10. The frequency, intensity and duration of headache diminish overtime. No obvious precipitating factor of headache was found. (See Supplementary table 1). No allergic symptoms or psychiatric symptoms presented in all cases.

### Laboratory investigation

Basic laboratory investigations were performed to exclude stroke mimics and to search for stroke risk factor at the time of presentation. None of our patients had significant abnormal laboratory findings. (Data not shown).

### CT and MRI

Fifteen patients (78.9%) had non-contrast CT scan of the brain as their initial imaging modality. Nineteen patients (100%) had MRI and 17 patients (89.5%) had MRI within the first 24 h of the presentation. All initial MRI were performed during the time of neurological deficits. Follow-up MRI were performed in all patients with median interval of 18 days (IQR 15–25).

All the CT scan and MRI were no evidence of intracranial bleeding or acute ischemic lesions.

### Vascular imaging

Seventeen patients underwent MR angiography without contrast injection within 24 h after symptom onset. Only 2 patients underwent CT angiography to avoid artifact from metal orthodontic brackets.

**Fig. 2:**
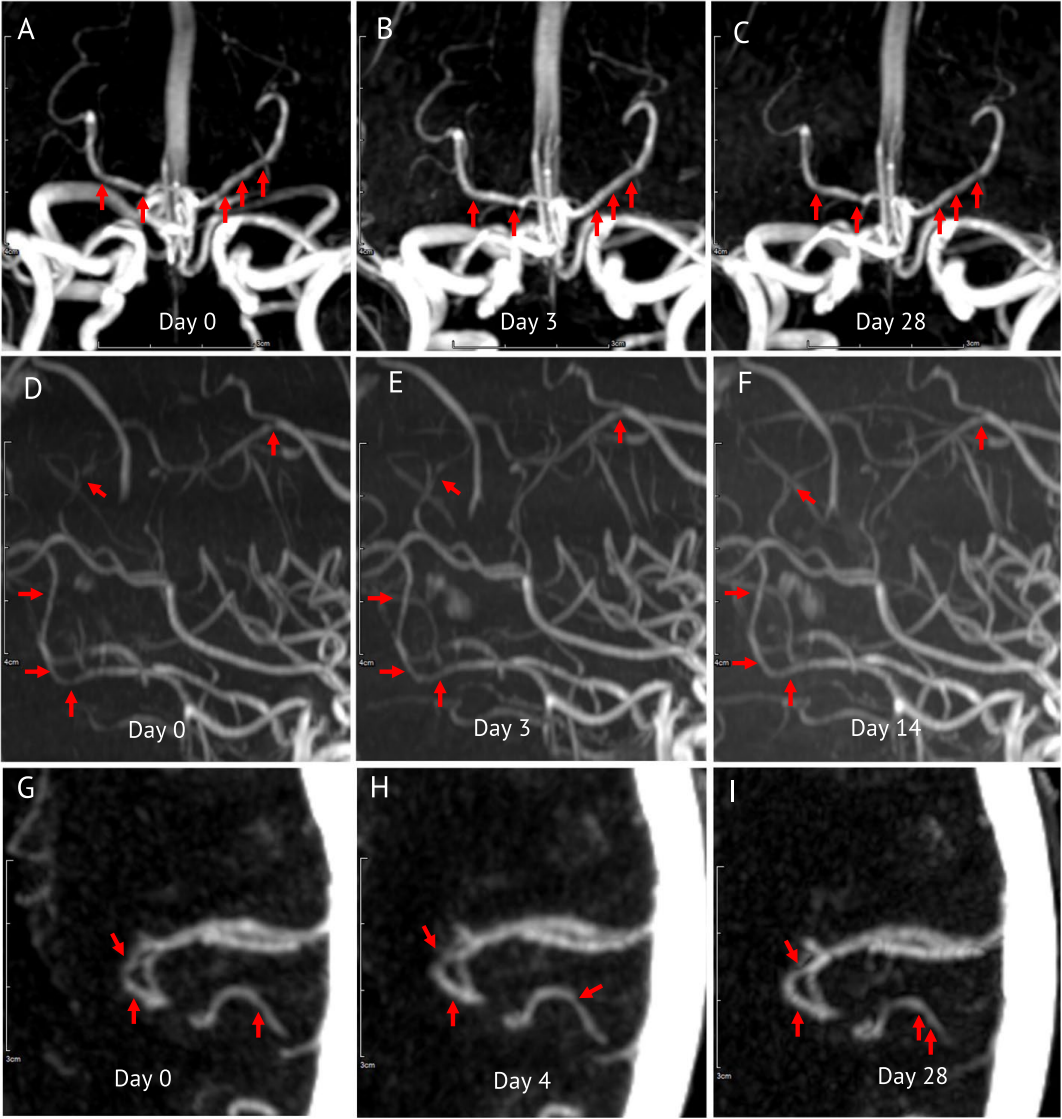
Vascular imaging features in patients with focal neurological symptoms after inactivated COVID-19 vaccination. Panel **A-C**, time-of-flight (TOF) magnetic resonance angiography (MRA) of the brain shows multifocal luminal irregularity and narrowing of bilateral posterior cerebral arteries (**A**) and subsequent imaging show reversion of the narrowing on day 3 (**B**) and day 28 (**C**). Panel D-F, TOF-MRA show multifocal narrowing of M3 segment of right middle cerebral artery (MCA) and focal narrowing of A3 segment of right anterior cerebral artery on day 0 (**D**) and later reversion on day 3 (**E**) and day 14 (**F**). Panel **G-I**, computed tomography angiography (CTA) of the brain shows focal narrowing of M2 segment of left MCA (**G**) and subsequent imaging show reversion of angiographic abnormality on day 4 (**H**) and new focal narrowing on day 28 (**I**)

Follow-up vascular imaging was performed in all patients to determined dynamic angiographic change with a median follow-up interval of 18 days (IQR 15,25 days, range 3–28 days). In 14 patients (73.7%) vascular imaging was performed 3 times and the other 5 had vascular imaging twice. Sixteen patients (84.2%) had no abnormalities on vascular imaging. However, interval changes of focal luminal narrowing were detected in 3 patients (15.8%) (Fig. 2). Notably, no medical risk factors including smoking and recreational drug used was reported from these patients.

### Treatment

All patients were initially evaluated under our stroke fast track protocol. However, due to the minor neurological deficits,the uncertainties of the etiology of symptoms and MRI failed to demonstrate evidence of brain infarction, intravenous thrombolysis was not given. Three main treatment strategies were applied in all cases: (1) Observe and reassure in 1 patient (5.3%) (2) oral calcium channel blocker such as nimodipine for 2 weeks in 16 patients (84.2%) (3) antiplatelet therapy such as aspirin in patients who had stroke-like symptom the initial onset and aspirin was discontinued after imaging followed up showed no evidence of brain infarction in 16 patients (84.2%). Symptomatic treatment including intravenous hydration and analgesics was routinely administration.

### The second dose of vaccination

Twelve patients (63%) receive the second dose of the same vaccine. Median interval between the first and the second dose was 21 days (IQR 21,21). New transient neurological symptoms were observed in 7 patients (58.3%). Six patients reported numbness, of these, 4 patients the symptoms occurred within 30 min after vaccination and 2 patients had the symptoms within 6 h. All patients reported the numbness confined in the same area of previous numbness after the first dose of vaccination. One patient reported single episode of thunderclap headache at day 3 after vaccination. No weakness was observed after the second dose of vaccination. (see Supplementary Fig. 1).

### Outcomes

The mean duration of clinical follow-up was 4 weeks. Recurrent focal neurological symptom was defined as recurrent episode of focal neurological symptoms after disappearing the previous symptoms. Recurrent headache defined as a present of new onset headache after disappearing the previous headache. Recurrent focal neurological symptoms were reported in 7 patients (36.8%) during 2 weeks after discharge. Recurrent unusual headache occurred later, at a median of 1 day after the initial symptoms. Recurrent headache occurred during the first week with median duration of 7 days (IQR 5,8), and completely disappeared within 2 weeks. For patients with recurrent symptoms, they had symptoms and deficit free in between.

Short term outcome at 2 weeks after discharge demonstrate favorable clinical outcome, an mRS score 0-1was found in 94.7%; an mRS score of 2–3 was found in 5.3% and no patients had severe deficits (mRS 4–5). Nearly half of patients still had neurological deficits at 2 weeks after discharge. Median headache free was at day 8 after vaccination (IQR 6,11).

At 1 month after the first dose of vaccination, mRS score 0–1 was found in 100%. Eight patients (42.1%) had persistent symptoms, 5 patients had numbness either hand, arm, or leg, 2 patients had tongue deviation, and 1 patient reported hand dexterity problem as difficulty writing. Delayed recovery of symptoms found in patient with sensory-motor symptom. (see Supplementary Fig. 2 and Supplementary Fig. 3).

## Discussion

We report a series of patients who developed unique neurological syndrome after inactivated COVID-19 vaccination. The characteristics of the syndrome include sudden onset of focal neurological deficits and recurrent headache without structural changes on brain imaging with good outcome in female predominance. The term immunization related focal neurological syndrome (IRFN) has been used to describe these patients. Some changes on cerebral vascular imaging were observed which may suggest the pathophysiology of vasoconstriction.

The main clinical presentation after vaccination was sudden onset focal neurological deficit and recurrent new-ever headache within 2 weeks after vaccination. All patients presented with focal neurological deficits on one side of the body which suggests unilateral cerebral dysfunction. The acuteness in nature could suggests vascular in origin. However, none of the patients developed ischemic lesions on initial and follow up MR imaging. Several mechanisms underlying this syndrome have been postulated (see Supplementary table 2). We propose 2 possible mechanism including 1) reversible cerebral vasoconstriction syndrome (RCVS) 2) migraine with aura.

In 3 cases in this series, irregularities and stenosis of the cerebral arteries were observed in the initial imaging and interval changes of the lesion sites were demonstrated. Although the MR angiographic studies in our cases were limited to non-contrast imaging, we used the same machine, methods, and sequences on the follow up imaging to avoid technical discrepancies. There were 2 patients who had CT angiography, transient irregularity of cerebral vessel was also depicted in one patient.

With the stroke like presentation and abnormalities seen on MRA in some cases, we postulated that focal neurological symptoms after vaccination might be a spectrum of RCVS. Stroke-like symptom is a clinical hallmark in the present series. Considering sudden onset of the symptoms raise the hypothesis that mechanism of the symptoms could derive from vascular in origin such as low cerebral blood flow from vasoconstriction, and recurrent new unusual headache over few days to 2 weeks after the initial onset in female predominance might supports a character of RCVS [6–8].

Generally, clinical characteristics of patients in the present series do not resemble typical RCVS. Transient neurological deficit from previous reports of RCVS usually delayed with a mean of 12 days after the initial headache onset [6] while in the present series, the symptom present early with a median of 55 min (IQR 21–661 min) after vaccination. However, the timeline of symptom is extremely quick for immunological vasculopathic manifestation to play a part. This acute manifestation within minutes to hours after vaccination raise the possibility that allergic reaction might play a role. Nonetheless, there was no common allergic symptoms presented after vaccination in this series. Thunderclap headache which is the main clinical presentation of with typical RCVS was found in only 1 case. Even so, existing evidence support that no thunderclap headache or unusual headache can present in RCVS patients [9]. Precipitating factors such as post-partum or vasoactive substance used is commonly found in RCVS [6] while no known provocative substance found in the present series except an exposure of COVID-19 vaccine.

Female predominant supports character of RCVS. Previous studies have shown that RCVS commonly affects female than male with female: male ratio range from 2.2:1 to 8.6:1 [6, 10, 11]. High proportion of female was also observed in this study with ratio 8.5:1. Existing evidence show that women with prior migraine have a higher frequency of having RCVS than men. Imbalance of female reproductive hormone is proposed to be a trigger of RCVS [8]. Notably, proportion of female of all participants who received vaccination in the period of interest is higher than male (68.3% vs 31.7% ratio 2.2:1) raise the concerning that female predominant might be occurred by chance.

Evidence of reversibility of angiographic abnormality within 12 weeks is one of criteria for diagnosis of RCVS. Although, most patients in the present series had no obvious abnormal vascular study for 4 weeks follow-up while 15.8% of patients had evidence of reversible luminal narrowing of cerebral vessels. We hypothesized that pathology might occurred at the small cortical branch or small penetrating artery therefore the imaging cannot detect the small dynamic change of the vessels. However, evidence of vascular change in this study is confounded by poor contrast and no contrast TOF images. Further investigation such as conventional cerebral angiography or digital subtraction angiography while patients still having symptoms might help clarify this hypothesis.

Transient unilateral weakness, spreading of sensory disturbance including perioral numbness followed by headache raise a hypothesis that migraine with aura or hemiplegic migraine might be an underlying mechanism of these symptoms after vaccination. Nearly half of patients in the present series had history of migraine and it is possible that vaccine might trigger migraine symptoms. Nonetheless, clinical characteristics of headache in the present series do not fulfil the criteria to diagnose migraine with aura for several reasons. First, characteristics of headache of most patients differ from previous headache and was not typical for migraine. Second, duration of neurological symptoms is longer than duration of migraine aura which should last for 5–60 min. Third, headache should follow the aura within 60 min but in the present series onset of headache varied from presenting at the initial onset to more than a week. Forth, no photophobia or phonophobia in the present series. Lastly, the duration of headache in most cases were less than 4 h [12].

Notably, headache is the commonly reported from COVID-19 vaccine recipients [13, 14]. Previous study reported headache started at 14.5 ± 21.6 h after received ChAdOx1 nCoV-19 vaccination and persisted for 16.3 ± 30.4 h and commonly located at forehead with dull aching in character [15]. Similarly, with BNT162b2 mRNA COVID-19 vaccine, headache begun 18 ± 27 h after vaccination, lasted for 14.2 ± 21.3 h,and most common located at forehead with pressing pain in character [16]. To our knowledge, this is the first case series describes character of headache related -inactivated COVID-19 vaccination, which is different from other COVID-19 vaccine in terms of recurrent long lasting until day 8 after initial onset and commonly located at vertex or occiput with band like in character. Despite of evidence support COVID-19 vaccination related with development of cerebral venous sinus thrombosis (CVST) in the setting of severe immune-mediated thrombotic thrombocytopenia especially woman in adenovirus vector-based SAR CoV2 vaccine [17, 18] and recent report in young healthy persons who received inactivated-virus COVID-19 vaccination [19]. However, abrupt onset of neurological symptoms with a median of 55 min of vaccination is atypical for CSVT that any developed symptoms usually occurred at 2 weeks [17] or at least 6 days after exposure to the vaccine [19].

Focal neurological deficit after inactive COVID-19 vaccination thought to be not a part of immunization stress-related response (ISRR) [20]. ISRR is used to describe symptoms that occur before or immediately occurred after immunization such as vasovagal reaction, dissociative neurological symptom reactions, including non-epileptic seizure [20]. However, no clinical symptom as describe in ISRR was observed in the present series.

According to an acute or abrupt onset of symptoms, possible mechanism beyond vascular in origin is allergic reaction, psychiatric symptoms, and functional neurological disorders or conversion disorder [14, 21, 22]. In the present series, no allergic symptoms were observed in all cases. Perioral numbness is also typically seen with psychiatric symptoms such as panic attacks, hyperventilation and anxiety; nevertheless, no psychiatric manifestation presented during period of observation and no patients had history of psychiatric disorder.

Function neurological disorders or conversion disorder can resemble any form of neurological deficits such as weakness, sensory symptom, abnormal movement, and seizure which onset usually acute, inconsistency in symptom and reversible with treatment include psychological intervention, psychodynamic therapy, or rehabilitation strategy in functional motor disorder [23]. The diagnosis is based on specific neurological examination or maneuver compatible with functional neurological disorders support by normal electroencephalogram, electrophysiologic test or laboratory tests in some phenotypes [23, 24]. Recent case reports have published functional neurological disorders trigger by SAR-Cov 2 vaccine [21, 25]. The diagnosis was made from positive Hoover’s sign in cases with motor weakness [25] and clear edge on facial midline in case with sensory loss [21]. Nonpyramidal distribution of paresis is one of less investigated positive sign in motor weakness, for functional neurological disorder, degree of weakness distributes equally in all muscle group. Furthermore, tongue deviate away to the paresis side supports functional neurological disorders [24]. In the present series, we observed pyramidal weakness pattern in a group of patients who had motor weakness and most patients with tongue deviation showed tongue deviate toward the paresis side. These findings are not qualified for functional neurological disorders. Future study on functional neurological disorders related SAR-Cov2 vaccine should be done with evidence support from electrophysiologic test, video electroencephalogram or laboratory test in selected phenotypes during the episode of symptoms to confirm a definite diagnosis. The outcome after the first vaccination was generally good (mRS0-1), however, 42.1% still had minor focal neurological deficit at 2 weeks. Unusual headache completely disappeared within 2 weeks after the first dose of vaccination. Neurological symptoms after the second shot of the vaccine can temporally occurred with mild symptoms.

This study did have some limitations. First, this is a small case series without a control group. Second, all the findings are observational only. Third, half of participants had history of migraine reported new different headache this could be a potential confounder. Forth, dynamic change of vascular narrowing could suggest RCVS, however, artifact issue with the non-contrasted and poor contrast cerebrovascular imaging should be concerned.

In summary, we report a series of patients who presented with transient focal neurological deficits after COVID-19 vaccination. The acute initial presentations and normal initial brain imaging may resemble acute ischemic stroke. However, the symptoms are usually mild with the majority of sensory abnormalities. Without thrombolytic treatment, all patients had favorable outcome and none of them developed infarction on follow up MRIs. Although this focal neurological syndrome can be alarming to both patients and physicians, it is a reversible process. We still strongly believe that COVID-19 vaccination is extremely important to stop the pandemic and decrease mortality from COVID-19 infection.

## Supplementary Information

**Additional file 1: Table S1.** Clinical characteristics, investigation, and outcome of entire cohort.

**Additional file 2: Table S2.** Postulate etiology of immunization related focal neurological syndrome (IFRN).

**Additional file 3: Figure S1.** Flow diagram of patients with neurological symptom after inactivated COVID-19 vaccine immunization.

**Additional file 4: Figure S2.** Recovery time after the first dose of inactivated COVID-19 vaccination stratified by symptoms.

**Additional file 5: Figure S3.** Kaplan-Meier plot estimates the progression of symptom-free survival in patient with neurological symptom after inactivated COVID-19 vaccination.

## Data Availability

All data generated or analyzed during this study are included in this published article and its supplementary information files.
