# Supplementary material for: Inactivated COVID-19 vaccine induced acute stroke-like focal neurologic symptoms: a case series
Source: BMC Neurol. 2022 Jun 7;22:210. doi: 10.1186/s12883-022-02739-6 (PMC9170873; doi:10.1186/s12883-022-02739-6)
Supplement: Supplementary file 1 — Additional file 1: Table S1. Clinical characteristics, investigation, and outcome of entire cohort. [file 12883_2022_2739_MOESM1_ESM.docx]

**Inactivated COVID-19 Vaccine Induced Acute Stroke-like Focal Neurologic Symptoms: A Case Series**

**Authors names:**

Duangnapa Roongpiboonsopit^1*^, Chichaya Nithisathienchai^1^,

Wasan Akarathanawat^2,3^, Krittanon Lertutsahakul^4^, Jarturon Tantivattana^4^,

Anand Viswanathan^5^, Nijasri Charnnarong Suwanwela^2,3,6^

**Affiliation:**

^1^ Division of Neurology, Department of Medicine, Faculty of Medicine, Naresuan University, Phitsanulok, Thailand

^2^ Division of Neurology, Department of Medicine, Faculty of Medicine, Chulalongkorn University, Bangkok, Thailand

^3^ Chulalongkorn Comprehensive Stroke Center, King Chulalongkorn Memorial Hospital, Bangkok, Thailand

^4^ Department of Radiology, Faculty of Medicine, Chulalongkorn University, Bangkok, Thailand

^5^ Department of Neurology, Harvard Medical School, Massachusetts General Hospital, Boston, MA, USA

^6^ Chula Neuroscience Center, King Chulalongkorn Memorial Hospital, Bangkok, Thailand

***Email address of corresponding author:** [Duangnapar@nu.ac.th](mailto:Duangnapar@nu.ac.th)

|  | | **Supplementary table 1** Clinical characteristics, investigation, and outcome of entire cohort. | | | | | | | | | | | | | | | | | | | | | | | | | | | | |  |  |  |  |
| --- | --- | --- | --- | --- | --- | --- | --- | --- | --- | --- | --- | --- | --- | --- | --- | --- | --- | --- | --- | --- | --- | --- | --- | --- | --- | --- | --- | --- | --- | --- | --- | --- | --- | --- |
| **Case** | **Age**  **/Sex** | | **History of allergy** | **Symptoms**  **onset after**  **VAC** | **Neurological symptom** | | **Neurological deficit** | | | **H/A** | | | | **H_x_ of migraine** | | | **H/A characteristics** | | | **H/A day** | **Vascular imaging /**  **abn. finding** | | | | | **Outcome**  **at 2^nd^ week** | **2^nd^ dose vaccine/**  **symptom** | | **Outcome at 4^th^ week** | | | | |  |
| 1 | 30/F | | None | 26 min | Sudden perioral and tongue numbness then L leg weakness | L hemiparesis (gr IV),  L hemiparesthesia, dysarthria,  tongue deviate to L | | | - | | | + | | | No H/A | | | No H/A | | | | MRA/+ | | mRS 1  (L hand, foot numbness) | | | - | | | mRS 1  (L hand numbness) | |  |  |  |
| 2 | 25/F | | None | 55 min | Sudden H/A then R hand numbness and weakness | R hemiparesis (gr IV),  R hemiparesthesia | | | * | | | - | | | Band like  at bitemporal radiate to orbit  PS 6/10,  last 2.5 hr,  peak 1 hr | | | 0,1,2,6 | | | | MRA/+ | | mRS 1  (Decrease dexterity  R hand) | | | - | | | mRS 1  (Decrease dexterity  R hand) | |  |  |  |
| 3 | 34/F | | None | 2.10 hr | Sudden L arm and  L leg numbness | No neurological deficit | | | +* | | | + | | | Band like  at occiput  PS 5/10,  last 30 min, peak 5 min | | | 1,4,5,6 | | | | MRA/- | | mRS 0 | | | +/ L leg numbness at  20 min after VAC | | | mRS 0 | |  |  |  |
| 4 | 29/F | | None | 2.09 hr | Sudden blurred vision, bilateral eyelid droop, rash at neck then L leg weakness | L hemiparesis (gr IV),  L hemiparesthesia | | | +* | | | - | | | Band like  at L temporal area PS 2/10  last 10 min | | | 3,6 | | | | MRA/- | | mRS 2  (L leg weakness) | | | - | | | mRS 1  (L leg numbness) | |  |  |  |
| 5 | 39/F | | None | 20 min | Sudden R arm numbness | No neurological deficit | | | +* | | | + | | | Troubling H/A at bitemporal  PS 4/10  last 8 hr  peak 30 min | | | 0,1 | | | | MRA/- | | mRS 0 | | | +/ R arm numbness at  20 min after VAC | | | mRS 0 | |  |  |  |
| 6 | 41/F | | Drug allergy | 5 min | Sudden numbness at lower lip then  R hand weakness | R hemiparesis (gr IV),  R hemiparesthesia  Tongue deviate to R | | | +* | | | - | | | Band like at bitemporal  with N/V  PS 8/10  last 2 min  peak 1 min | | | 1,2,4,5,6 | | | | MRA/- | | mRS 1  (Tongue deviation) | | | - | | | mRS 1  (Tongue deviation) | |  |  |  |
|  |  | |  |  |  |  | | |  | | |  | | |  | | |  | | | |  | |  | | |  | | |  | |  |  |  |
|  | | **eTable 1**-*continue* | | | | | | | | | | | | | | | | | | | | | | | | | | | | | | |  |  |
| **Case** | **Age**  **/Sex** | | **History of allergy** | **Symptoms onset after VAC** | **Neurological symptom** | | | **Neurological deficit** | | | **H/A** | | **H_x_ of migraine** | | | **H/A characteristics** | | | **H/A day** | | | | **Vascular imaging /abn. finding** | | **Outcome at 2^nd^ week** | | | **2^nd^ dose vaccine/**  **symptom** | **Outcome at 4^th^ week** | | | | | |
| 7 | 28/F | | None | 7 min | Sudden H/A and L side numbness | | | No neurological deficit | | | +* | | + | | | Band like at bitemporal  PS 8/10 last 3 hr  peak 30 min | | | 1,2,4,5,6 | | | | CTA/- | | mRS 0 | | | +/thunder-clap H/A  at day 3 after VAC | mRS 0 | | | | | |
| 8 | 46/F | | Drug allergy | 10.17 hr | Sudden H/A during sleep then L leg numbness | | | Decrease PPS L dorsum of foot | | | +* | | - | | | Band like  at occiput  PS 5/10 last 1 hr  peak 10 min | | | 0,3,4 | | | | MRA/- | | mRS 0 | | | + | mRS 0 | | | | | |
| 9 | 23/F | | None | 44 min | Sudden L arm numbness | | | L hemiparesis (gr IV), decrease PPS L arm | | | +* | | - | | | Band like  at bitemporal  PS 5/10  last 45 min  peak 30 min | | | 0,4,5 | | | | MRA/- | | mRS 1  (LhemiparesisL arm, L foot numbness) | | | - | mRS 0 | | | | | |
| 10 | 41/F | | None | 10 min | Sudden H/A, vertigo, nausea vomiting, ataxia to the L and L hand weakness | | | L hemiparesis (gr III),  L hemiparesis,  tongue deviate to R | | | + | | + | | | Band like  at bitemporal  PS 7/10  last 1 hr  peak 45 min | | | 0,2,3,4 | | | | MRA/- | | mRS 0 | | | +/ L foot numbness  at 2.46 hr after VAC | mRS 1  (L foot numbness) | | | | | |
| 11 | 41/F | | Drug allergy/Food allergy | 21 min | Sudden perioral numbness R upper lip then R hemiparesis | | | R hemiparesis,  R hemiparesthesia | | | +* | | - | | | Band like  at occiput  PS 3/10  last 60 min  peak 2 min | | | 0,1,2 | | | | MRA/- | | mRS 0 | | | +/ R hand numbness  at 10 min after VAC | mRS 0 | | | | | |
| 12 | 31/F | | None | 8 min | Blurred vision both eye, vertigo, nausea and vomiting, ataxia, thunderclap H/A then L hand numbness | | | Spastic dysarthria, decrease R nasolabial fold, tongue deviate to L, triparesis (R upper Ext gr IV, L upper Ext gr III, L lower Ext gr IV)  decrease PPS L arm | | | +* | | - | | | Generalized sharp shooting H/A  PS 8/10  last 30 min  Peak 1 min | | | 0,2,5,6,7 | | | | CTA/+ | | mRS 0 | | | - | mRS 0 | | | | | |
|  |  | |  |  |  | | |  | | |  | |  | | |  | | |  | | | |  | |  | | |  |  | | | | | |
|  | | **eTable 1**-*continue* | | | | | | | | | | | | | | | | | | | | | | | | | | | | | | |  |  |
| **Case** | **Age**  **/Sex** | | **History of allergy** | **Symptoms onset after VAC** | **Neurological symptom** | **Neurological deficit** | | | **H/A** | | | **H_x_ of migraine** | | | **H/A characteristics** | | | **H/A day** | | | | **Vascular imaging /abn. finding** | | **Outcome at 2^nd^ week** | | | **2^nd^ dose vaccine/**  **symptom** | | | **Outcome at 4^th^ week** | |  |  |  |
| 13 | 29/F | | None | 3.24 hr | Sudden L leg weakness and numbness | Monoparesis L leg (gr IV), decrease PPS L leg | | | +* | | | + | | | Dull aching H/A  at vertex  PS 5/10  last 15 min  peak 5 min | | | 3,4,6 | | | | MRA/- | | mRS 0 | | | +/ L leg numbness persists  since the 1^st^ dose of VAC | | | mRS 1  (L leg numbness) | |  |  |  |
| 14 | 35/M | | None | 48 min | Sudden R leg weakness and numbness | R leg monoparesis (gr IV), decrease PPS R foot | | | + | | | + | | | Troubling H/A at R temporal  PS 6/10  last 2 hr  Peak 30 min | | | 1,2,5 | | | | MRA/- | | mRS 0 | | | + | | | mRS 0 | |  |  |  |
| 15 | 33/F | | None | 11.45 hr | Sudden R leg numbness | Decrease PPS R leg | | | +* | | | - | | | Band like  at bitemporal  PS 5/10 last 4 hr  peak 1 hr | | | 5,6 | | | | MRA/- | | mRS 0 | | | +/ R leg numbness  at 15 min after VAC | | | mRS 0 | |  |  |  |
| 16 | 45/F | | None | 1 day  1.10 hr | Sudden severe H/A, N/V | No neurological deficit | | | +* | | | - | | | Band like  at occiput  PS 7/10  last 26 hr | | | 1,2 | | | | MRA/- | | mRS 0 | | | + | | | mRS 0 | |  |  |  |
| 17 | 24/F | | None | 5 days  13.10 hr | Sudden onset R arm and R leg numbness | Decrease PPS R arm and leg, tongue deviate to R | | | + | | | + | | | Troubling H/A at R temporal  PS 4/10  last 1 hr  Peak 15 min | | | 8 | | | | MRA/- | | mRS 1  (Tongue deviation) | | | - | | | mRS 1  (Tongue deviation) | |  |  |  |
| 18 | 27/F | | None | 4 days  14.30 hr | Sudden onset R hand weakness and R side numbness | R hemiparesthesia | | | +* | | | + | | | Troubling H/A at occiput  PS 5/10  last 3 hr  Peak 10 min | | | 10,11,12 | | | | MRA/- | | mRS 0 | | | +/ R side numbness  at 3.5 hr after VAC | | | mRS 0 | |  |  |  |
|  |  | |  |  |  |  | | |  | | |  | | |  | | |  | | | |  | |  | | |  | | |  | |  |  |  |
|  |  | |  |  |  |  | | |  | | |  | | |  | | |  | | | |  | |  | | |  | | |  | |  |  |  |
|  | | **eTable 1**-*continue* | | | | | | | | | | | | | | | | | | | | | | | | | | | | | | |  |  |
| **Case** | **Age**  **/Sex** | | **History of allergy** | **Symptoms onset after VAC** | **Neurological symptom** | **Neurological deficit** | | | **H/A** | | | **H_x_ of migraine** | | | **H/A characteristics** | | | **H/A day** | | | | **Vascular imaging /abn. finding** | | **Outcome at 2^nd^ week** | | | **2^nd^ dose vaccine/**  **symptom** | | | **Outcome at 4^th^ week** | |  |  |  |
| 19 | 45/M | | none | 4 days  12.30 hr | Sudden onset L leg numbness | Decrease PPS L leg | | | - | | | - | | | No H/A | | | NA | | | | MRA/- | | mRS 1  (L face, trunk, arm numbness) | | | + | | | mRS 1  (L arm numbness) | |  |  |  |

“* “New headache”. The patients reported that the character of headache was new and district from the previous experience.”

No patients reported history of migraine with aura.

Abbreviation: abn. : abnormal, CTA: computed tomography angiography, Ext: extremity, gr: grade, H/A: headache, H_x:_ history, L: left, MRA: magnetic resonance angiography, mRS: modified rankin scale, N/V: nausea vomiting, PPS: pinprick sensation, PS: pain score, R: right, VAC : vaccination
