# Supplementary material for: Inactivated COVID-19 vaccine induced acute stroke-like focal neurologic symptoms: a case series
Source: BMC Neurol. 2022 Jun 7;22:210. doi: 10.1186/s12883-022-02739-6 (PMC9170873; doi:10.1186/s12883-022-02739-6)
Supplement: Supplementary file 2 — Additional file 2: Table S2. Postulate etiology of immunization related focal neurological syndrome (IFRN). [file 12883_2022_2739_MOESM2_ESM.docx]

**Inactivated COVID-19 Vaccine Induced Acute Stroke-like Focal Neurologic Symptoms: A Case Series**

**Authors names:**

Duangnapa Roongpiboonsopit^1*^, Chichaya Nithisathienchai^1^,

Wasan Akarathanawat^2,3^, Krittanon Lertutsahakul^4^, Jarturon Tantivattana^4^,

Anand Viswanathan^5^, Nijasri Charnnarong Suwanwela^2,3,6^

**Affiliation:**

^1^ Division of Neurology, Department of Medicine, Faculty of Medicine, Naresuan University, Phitsanulok, Thailand

^2^ Division of Neurology, Department of Medicine, Faculty of Medicine, Chulalongkorn University, Bangkok, Thailand

^3^ Chulalongkorn Comprehensive Stroke Center, King Chulalongkorn Memorial Hospital, Bangkok, Thailand

^4^ Department of Radiology, Faculty of Medicine, Chulalongkorn University, Bangkok, Thailand

^5^ Department of Neurology, Harvard Medical School, Massachusetts General Hospital, Boston, MA, USA

^6^ Chula Neuroscience Center, King Chulalongkorn Memorial Hospital, Bangkok, Thailand

***Email address of corresponding author:** [Duangnapar@nu.ac.th](mailto:Duangnapar@nu.ac.th)

**Supplementary table 2** Postulate etiology of immunization related focal neurological syndrome (IFRN)

**Character of IFRN**: Sudden onset focal neurological deficits, recurrent unusual headache, no structural changes on initial brain imaging, good outcome and female predominance.

| **Postulated etiology** | **Factor favoring** | **Factor against** |
| --- | --- | --- |
| Reversible Cerebral Vasoconstriction Syndrome (RCVS) | Acuteness in nature could suggests vascular in origin | Neurological deficits present early after vaccination (55 min) while RCVS usually delayed (12 days) after the initial headache onset |
|  | Evidence of transient irregularity of cerebral vessel. | Most character of headache is not thunderclap headache |
|  | Recurrent Unusual headache |  |
|  | Female predominance |  |
| Migraine (migraine with aura or hemiplegic migraine) | Spreading of sensory disturbance | New usual headache, not typical for migraine |
|  | Perioral numbness | Duration of neurological symptoms is longer than duration of migraine aura which should last for 5-60 minutes |
|  | Transient unilateral weakness | Duration of headache is longer than typical migraine aura |
|  | History of migraine in nearly half patients | No photophobia or phonophobia |
| **Other possible etiology** | **Factor favoring** | **Factor against** |
| Immunization stress-related response (ISRR) | Symptoms occur immediately after immunization | No symptoms describe in ISRR including vasovagal reaction, dissociative neurological reaction such as non-epileptic seizure was observed on this case series |
|  | Reversible symptoms without structural change on neuroimaging |  |
| Allergic reaction | Symptoms occur immediately after immunization | No common allergic symptoms were observed |
| Psychiatric symptoms | Perioral numbness is also typically seen in panic attacks, hyperventilation syndrome and anxiety | No psychiatric manifestation presented during period of observation |
|  | Symptoms occur immediately after immunization | No history of psychiatric disorders |
| Functional neurological disorders | Resemble any form of neurological deficits | Pyramidal weakness pattern |
|  | Acute onset | Tongue deviate toward the paresis side in most cases |
|  | No structural lesion on brain imaging |  |
| Cerebral venous sinus thrombosis | Headache after immunization | Symptoms rapidly occurred after vaccination |
|  | Female predominance |  |
