# Supplementary material for: Inactivated COVID-19 vaccine induced acute stroke-like focal neurologic symptoms: a case series
Source: BMC Neurol. 2022 Jun 7;22:210. doi: 10.1186/s12883-022-02739-6 (PMC9170873; doi:10.1186/s12883-022-02739-6)

**Inactivated COVID-19 Vaccine Induced Acute Stroke-like Focal Neurologic Symptoms: A Case Series**

**Authors names:**

Duangnapa Roongpiboonsopit^1*^, Chichaya Nithisathienchai^1^,

Wasan Akarathanawat^2,3^, Krittanon Lertutsahakul^4^, Jarturon Tantivattana^4^,

Anand Viswanathan^5^, Nijasri Charnnarong Suwanwela^2,3,6^

**Affiliation:**

^1^ Division of Neurology, Department of Medicine, Faculty of Medicine, Naresuan University, Phitsanulok, Thailand

^2^ Division of Neurology, Department of Medicine, Faculty of Medicine, Chulalongkorn University, Bangkok, Thailand

^3^ Chulalongkorn Comprehensive Stroke Center, King Chulalongkorn Memorial Hospital, Bangkok, Thailand

^4^ Department of Radiology, Faculty of Medicine, Chulalongkorn University, Bangkok, Thailand

^5^ Department of Neurology, Harvard Medical School, Massachusetts General Hospital, Boston, MA, USA

^6^ Chula Neuroscience Center, King Chulalongkorn Memorial Hospital, Bangkok, Thailand

***Email address of corresponding author:** [Duangnapar@nu.ac.th](mailto:Duangnapar@nu.ac.th)

**Supplementary figure 1** Flow diagram of patients with neurological symptom after inactivated COVID-19 vaccine immunization


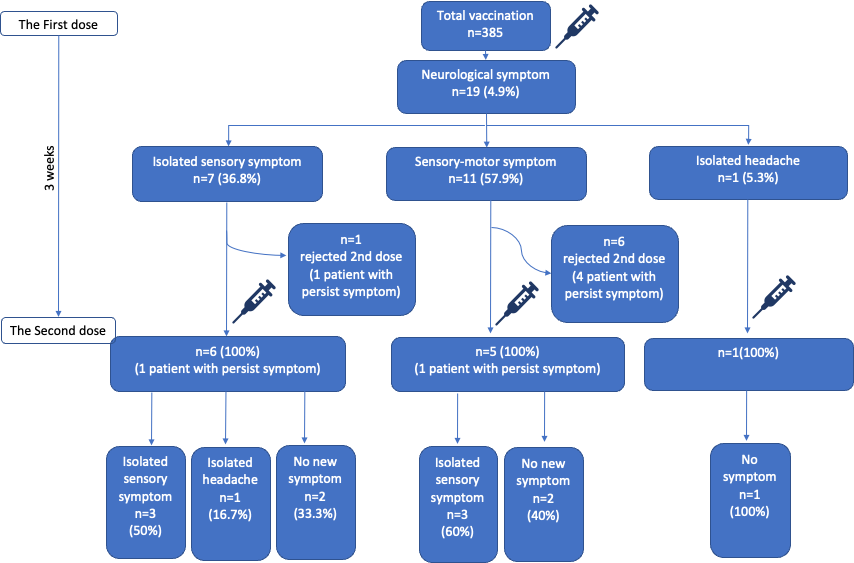

Supplement: Supplementary file 3 — Additional file 3: Figure S1. Flow diagram of patients with neurological symptom after inactivated COVID-19 vaccine immunization. [file 12883_2022_2739_MOESM3_ESM.docx]
